# Oocyte-triggered dimerization of sperm IZUMO1 promotes sperm–egg fusion in mice

**DOI:** 10.1038/ncomms9858

**Published:** 2015-11-16

**Authors:** Naokazu Inoue, Yoshihisa Hagihara, Danelle Wright, Takahisa Suzuki, Ikuo Wada

**Affiliations:** 1 Department of Cell Science, Institutes for Biomedical Sciences, School of Medicine, Fukushima Medical University, 1 Hikarigaoka, Fukushima City, Fukushima 960-1295, Japan; 2 National Institute of Advanced Industrial Science and Technology, 1-8-31 Midorigaoka, Ikeda, Osaka 563-8577, Japan

## Abstract

Sperm–egg fusion is indispensable for completing mammalian fertilization. Although the underlying molecular mechanisms are poorly understood, requirement of two spermatozoon factors, IZUMO1 and SPACA6, and two oocyte factors, CD9 and the IZUMO1 counter-receptor JUNO, has been proven by gene disruption, and the binding of cells to an oocyte can be reconstituted by ectopic expression of IZUMO1. Here we demonstrate that robust IZUMO1-dependent adhesion of sperm with an oocyte accompanies the dimerization of IZUMO1. Despite the intrinsic dimeric property of its N-terminal region, IZUMO1 is monomeric in spermatozoa. Interestingly, JUNO associates with monomeric IZUMO1, which is then quickly removed as tight adhesion of the two cells is subsequently established. We therefore propose that global structural rearrangement of IZUMO1 occurs on JUNO recognition and that this rearrangement may then initiate force generation to overcome repulsion between the juxtaposing membranes, through an unidentified receptor on the egg.

## Abstract (web summary)

Sperm-egg fusion is facilitated by the sperm-specific receptor IZUMO1 and the egg-specific counter-receptor JUNO. Here Inoue *et al.* show that JUNO first binds to monomeric IZUMO1; IZUMO1 then dimerizes and excludes JUNO from the sperm-egg interface, suggesting the existence of a IZUMO1 dimer receptor.

## Graphical abstract

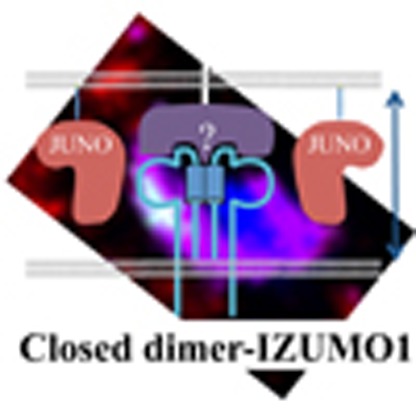

Among the various steps of fertilization, gamete membrane fusion must be an extremely robust and precise mechanism, as it is the climax of fertilization. Through analysis of genetically modified mice, CD9 on the egg and IZUMO1 on the spermatozoon have been identified as essential factors in fusion^1^,^2^,^3^,^4^. In 2014, the IZUMO1 counter-receptor JUNO was discovered on the egg surface^5^. Moreover, SPACA6 on spermatozoa was found to also participate in the process^6^. Mice lacking either factor are sterile because of the failure of sperm–egg fusion. However, the exact molecular mechanisms leading to gamete membrane fusion remain elusive^7^.

IZUMO1 is a type I membrane protein composed of an immunoglobulin-like domain in the extracellular region, a single membrane-spanning domain, and a short cytoplasmic tail^4^. We previously showed that this acrosomal protein is translocated to the equatorial segment of the sperm surface after the acrosome reaction^8^. The structure of the extracellular domain is apparently critical for its function, while the intracellular region contains only one amino acid in porcine IZUMO1 (ref. 9). This domain contains a conserved sequence motif, the Izumo domain, where a cluster of eight cysteines—C-X(2)-C-X(106,108)-C-X(3,4)-C-X(9)-C-X(2)-C-X(6)-C-X(4,5)-C is found^10^. The immunoglobulin-like domain contains a well-conserved *N*-glycan, and we showed that it protects IZUMO1 from degradation in the cauda epididymis^11^.

Recently, we specified the critical domain of IZUMO1 for sperm–egg fusion and clarified its function by epitope mapping fusion-inhibiting monoclonal antibodies and by a series of biophysical measurements^12^. This fragment, including the potential functional site, binds directly to zona-free eggs and has fusion-inhibitory activities in an *in vitro* fertilization system. The fragment is composed of an N-terminal unfolded structure and a C-terminal ellipsoidal helical dimer. We were also successful in establishing an *in vitro* system in which cultured cells, such as COS-7, become adhesion-competent towards oocytes by expressing the *Izumo1* gene.

This study focuses on the mechanism of the structural changes of IZUMO1 that occur at the moment of fusion, which can only be viewed in a cultured cell zona-free oocyte-binding system, as it is incapable of proceeding to fusion. By using newly produced IZUMO1 monoclonal antibodies, bimolecular fluorescence complementation (BiFC), and a photon-counting histogram (PCH), we found that dimerization of IZUMO1 can occur at the adhesive surface between cultured cells or between spermatozoa and an oocyte, and appears to be critical for the tight binding. The analyses also demonstrated that JUNO no longer existed at the interface, strongly suggesting the presence of an alternative egg receptor other than JUNO.

## Results

### Characterization of new IZUMO1 antibodies

Until now, to elucidate the molecular mechanism of sperm–egg fusion, we focused on fusion factor IZUMO1 (ref. 4). In our latest paper^12^, we found that the N-terminal region, Asp5-Leu113, of this protein was essential in expressing fusion activity^12^. This fragment consists of two distinguished structures, the unstructured domain at the N terminus (Asp5-Ala56) and a helical dimer at the C terminus (Val57-Leu113). In addition, we established an *in vitro* system that mimics sperm–egg adhesion by using *Izumo1-*expressing cultured cells and oocytes^12^. Utilizing this system, we analysed the molecular mechanism(s) that couples adhesion with fusion.

In this work, first, we produced two novel monoclonal antibodies (Mab17 and Mab18) against the IZUMO1_5–113_ fragment. In *in vitro* fertilization of mouse gametes, Mab17 showed potent inhibition, whereas Mab18 did not disturb fertilization (Supplementary Fig. 1a,b). In fact, many spermatozoa accumulated in the perivitelline space 24 h after being treated with Mab17 (Supplementary Fig. 1b, upper middle photo). The analyses using surface plasmon resonance (SPR; Supplementary Fig. 1c,d) indicated that Mab17 recognized the α-helical IZUMO1_57–113_ and that the Mab18 epitope resided in the N-terminal domain IZUMO1_26–46_. Because the Mab17 epitope was destroyed by helical breakers (5-113-Pro in Supplementary Fig. 1d), this antibody most likely recognizes the α-helical structure. A schematic diagram of the binding sites of IZUMO1 monoclonal antibodies in this study is shown in Supplementary Fig. 1e.

**Figure 1:**
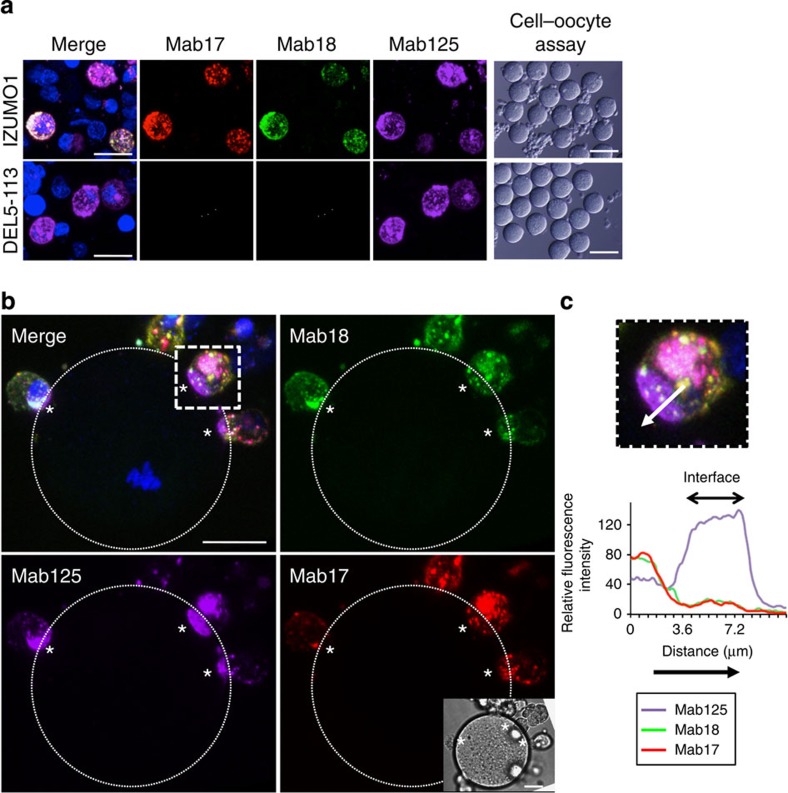
Distinct profiles of monoclonal antibodies recognizing IZUMO1 in cell–oocyte assay. (**a**) Immunostaining and cell–oocyte assay of a COS-7 cell expressing *Izumo1* and DEL5-113. COS-7 cells that were transfected with mouse IZUMO1 and the truncated DEL5-113 cDNA were stained with Mab17-Alexa546 (red), Mab18-Alexa488 (green) and Mab125-Alexa647 (magenta) simultaneously after dissociation by 10 mM EDTA containing PBS, as described in Methods. The final concentration of all antibodies added was at 0.5 μg ml^−1^. Scale bars, 20 μm. Right photographs show the transmission images of the cell–oocyte assay. Scale bars, 100 μm. (**b**) Cell–oocyte assay with IZUMO1-specific antibodies. *Izumo1*-expressing COS-7 cells and oocytes were incubated for 2 h at 37 °C with the monoclonal antibodies in **a** (at the same concentrations). Inset shows differential interference contrast (DIC) image of the centre of the Z-stacks. Some attached COS-7 cells are out of focus. The adherent surface of COS-7 cells to the oocyte is indicated by asterisks. Nuclei were stained with Hoechst 33342. Scale bar, 20 μm. The outline of the oocyte is marked by dotted lines. (**c**) The relative fluorescence intensities at the interface. The × 2 magnified image of the white dashed box in **b** is shown. The relative fluorescence intensity in the white arrow line of the dashed box in **c** was analysed with ImageJ. The fluorescence intensities of Mab17-Alexa546, Mab18-Alexa488 and Mab125-Alexa647 are indicated in red, green and purple lines, respectively. The arrow indicates a measurement direction.

When IZUMO1 was transiently expressed in COS-7 cells, all antibodies reacted only with *Izumo1*-expressing cells (Fig. 1a). In the truncated mutant lacking Asp5-Leu113 (DEL5-113), neither of the new antibodies showed any reactivity as expected, whereas it was well recognized by the non-inhibitory antibody (Mab125)^12^ (Fig. 1a), along with the assumed epitope Pro234-Arg298 (Supplementary Fig. 1e). Expression of the truncated mutant in COS-7 cells had no effect on egg binding (Fig. 1a). Consistent with the above *in vitro* fertilization results, Mab17, but not Mab18, completely inhibited the COS-7 cell–egg association at 10 μg ml^−1^ as well as *in vitro* fertilization (Supplementary Fig. 1b). We thought that these results might stress the importance of the helical region in the function of IZUMO_57–113_.[Fig f2][Fig f3]

### New monoclonal antibodies could not bind to the interface

By using these monoclonal antibodies, we next analysed the distribution of epitopes of each antibody in *Izumo1-*expressing COS-7 cells with an oocyte. In this cell–oocyte assay, the average number of attached cells on the oocyte surface was 5.48±0.17 (Fig. 4g; 60 oocytes, *n*=3, mean±s.e.m.). Regarding the non-inhibitory antibody Mab125, we previously reported that IZUMO1 was clustered at the interface of the egg–COS-7 cells^12^. Surprisingly, neither of the newly produced antibodies showed binding to the interface, whereas Mab125 tended to recognize IZUMO1 more on the interface than on the rest of the surface (Fig. 1b; images from different angles are shown in Supplementary Fig. 5a). Figure 1c is the quantification of the cell image (the white dashed box in Fig. 1b), indicating that reactivity of Mab17/Mab18 was almost negative in the interface that was intensely labelled by Mab125 (Fig. 1c). These properties resembled the OBF13 antibody, which was used to identify IZUMO1 (ref. 4), and recognized IZUMO1_5–113_ (ref. 12). When OBF13 was employed, adhesion of COS-7 cells to eggs was inhibited at 10 μg ml^−1^ (Supplementary Fig. 1f). Furthermore, the staining pattern of OBF13 at 0.2 μg ml^−1^, which allowed the binding, almost overlapped with Mab125 in the non-adherent cells (Supplementary Fig. 1g, upper panels). The interface of COS-7 cells with the egg was intensively stained with Mab125 (Supplementary Fig. 1g, asterisks) as well as the membrane fragments of COS-7 cells (Supplementary Fig. 1g, arrows), which could have been caused by pipetting. However, no OBF13 reaction was observed on the interface (Supplementary Fig. 1g, lower panels).

These observations are consistent with the concept that IZUMO1 is clustered at the interface. We noticed that this property was distinct from SPACA6, the new fusion factor recently identified on the sperm side^6^. When the localization of the fluorescent fusion proteins was compared in oocyte–COS-7 cell assays expressing both proteins, SPACA6 did not accumulate in the interface, unlike IZUMO1-mCherry (Supplementary Fig. 1h), suggesting that SPACA6 may function in the later stages of IZUMO1 binding. In fact, solely *Spaca6*-expressing COS-7 cells did not adhere to the egg surface.

### Dimerization of IZUMO1 selectively occurs at the interface

**Figure 2 f2:**
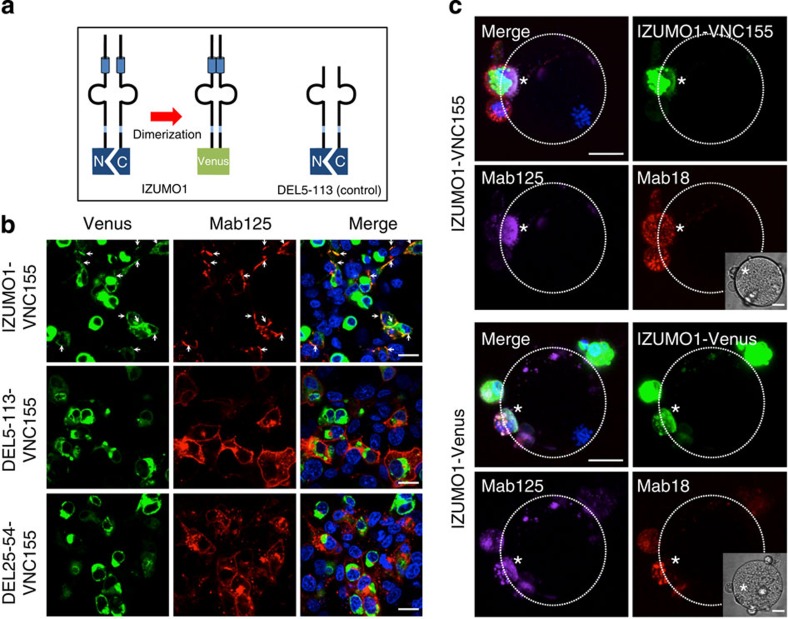
Oligomerization of IZUMO1 as revealed by BiFC analysis. (**a**) The schematic diagram of the BiFC analysis. When dimerization occurs, the non-fluorescent fragments VN155 and VC155 are brought in close vicinity to form a fluorophore. (**b**) BiFC analysis on cultured cells. BiFC detected at the surface of COS-7 cells expressing *Izumo1* in addition to intracellular structures. COS-7 cells were co-transfected with either full-length IZUMO1, Asp5-Leu113 (DEL5-113) or Pro5-Leu54 (DEL5-54)-truncated IZUMO1, each of which was fused to both VN155 and VC155 (IZUMO1-VNC, green). Before observation, the live cells were stained with Mab125-Alexa546 (red) at 0.5 μg ml^−1^. Although the complementation signal was observed in intracellular structures of all experiments, only the full-length IZUMO1 rendered fluorescence on the cell surface (arrows), which was marked by Mab125-Alexa546. (**c**) BiFC analysis in cell–oocyte assay. In combination with the BiFC analysis, cell–oocyte assays were performed. The asterisks indicate adhered cells. IZUMO1 was stained with both of Mab18-Alexa546 (red) at 0.5 μg ml^−1^ and Mab125-Alexa647 (magenta) at 0.5 μg ml^−1^. The green fluorescence image by BiFC (top) or Venus (bottom) was captured simultaneously (green). The cell next to an oocyte-adhered cell (asterisk) bound to the COS-7 cell but not the oocyte. Inset shows middle DIC image among Z-stacks. Nuclei were stained with Hoechst 33342. Scale bar, 20 μm. The outline of the oocyte is marked by dotted lines.

We then reasoned that the higher structural configuration of IZUMO1_5–113_ may be altered at the interface between COS-7 cells and the egg so that both Mab17 and Mab18 showed little reactivity to the adherent domain. We previously demonstrated that IZUMO1_5–113_ formed a dimer, and thus examined the possibility that the IZUMO1_5–113_ region may alter the oligomeric structure on binding to an egg. To this end, we employed BiFC to visualize dimerization of IZUMO1 in locally sites. The BiFC assay is based on the reconstitution of a fluorophore when two complementary non-fluorescent Venus-truncated fragments such as VN155/VC155 are brought together by a pair of the fused proteins in close proximity^13^. We designed IZUMO1-VN155 and -VC155 to emit green fluorescence only when they form a dimer (Fig. 2a). For simplicity, we designated VNC155 to indicate both VN155 and VC155.

We constructed a set of either IZUMO1 full-length or truncated mutants fused to both VN155 and VC155 and expressed the set, VNC155, simultaneously in COS-7 cells. When the truncated IZUMO1 lacking Asp5-Leu113 or Pro25-Leu54, designated as DEL5-113-VNC155 or DEL25-54-VNC155, respectively, were expressed, both mutants reached the cell surface. This was shown by Mab125-Alexa546 staining without permeabilization (Fig. 2b). However, the Venus-derived autofluorescence was not found with the cell surface signal. The intracellular Venus signal appeared to represent a population of improperly folded aggregates, which were most likely trapped by the quality-control mechanisms of the early secretory pathway. The plasma membrane portion of IZUMO1-Venus in COS-7 cells did indeed show relatively rapid recovery after photobleaching (maximum recovery rate: 53.0%). On the other hand, the recovery was almost completely abolished in IZUMO1-VNC155 that accumulated in a distinct juxtanuclear area (maximum recovery rate: 11.7%) as well as in the endoplasmic reticulum (ER)-like structure of IZUMO1-Venus in COS-7 cells (recovery rate: 5.5%). This suggests that the misfolded IZUMO1-VNC155 protein forms aggregates and becomes trapped inside the ER, preventing protein trafficking (Supplementary Fig. 2a). Contrary to this, in cells expressing *Izumo1-VNC155*, a strong Mab125 signal was found in the polarized region of the cell surface, and the complementation-derived Venus fluorescence was also found at that region (Fig. 2b, arrows). These results are consistent with our previous observation showing the dimeric property of IZUMO1 through oligomerization of the N-terminus segment composed of the helical domain, and the unstructured N-terminus region. When COS-7 cells expressing IZUMO1-VNC155 bound to oocytes were examined, Venus fluorescence appeared at the interface as marked by Mab125 (Fig. 2c, upper panels, asterisk; images from different angles are shown in Supplementary Fig. 5b). Similarly, when 293T cells expressing the same fusion proteins were used, essentially the same results were obtained (Supplementary Fig. 2b,c).

IZUMO1 fused to intact Venus (Fig. 2c, lower panels) or cysteine free strongly enhanced green fluorescent protein 2 (cfSGFP2) (Supplementary Fig. 2d)^14^, however, showed rather uniform distribution including both the interface and punctate structures on the rest of the surface.

### Monomer form of IZUMO1 binds to JUNO

**Figure 3 f3:**
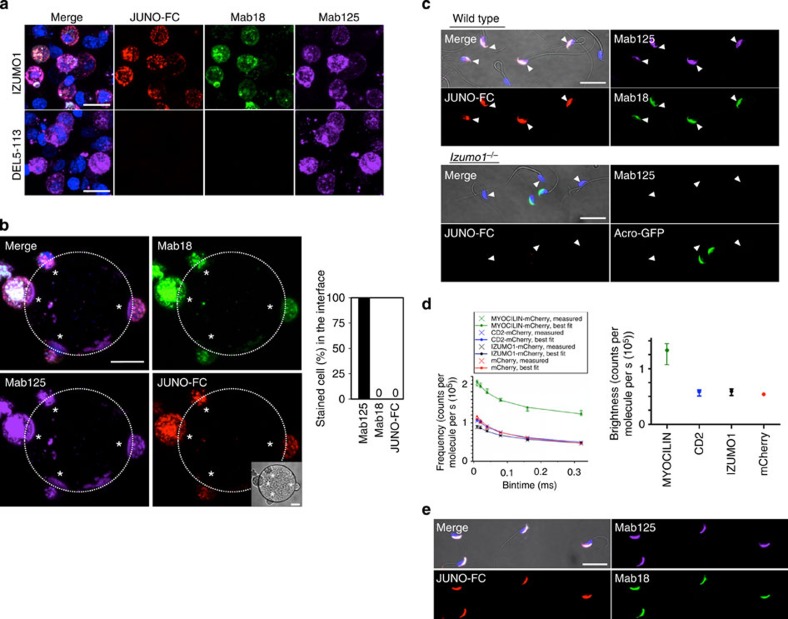
Recombinant JUNO binds to spermatozoon where IZUMO1 is monomeric. (**a**) Binding of recombinant JUNO to IZUMO1. The JUNO-FC fusion protein labelled with α-mouse IgG antibodies-Alexa546 selectively bound to IZUMO1-expressing cells (red). Simultaneously, they were incubated with Mab18-Alexa488 (green) and Mab125-Alexa647 (magenta). (**b**) Cell–oocyte assay with recombinant JUNO. The same experiment was carried out with oocytes. The interface was devoid of JUNO-FC signal (asterisks). The right graph shows the percentage of stained cells in the cell–oocyte interface. Ten oocytes and fifty-seven attached cells were investigated in this analysis. The image of the oocyte is marked by dotted lines. Inset shows middle DIC image among Z-stacks. (**c**) Immunostaining of wild-type and IZUMO1-null spermatozoa with JUNO-FC. Wild-type fresh spermatozoa were incubated for 2 h in TYH medium with JUNO-FC (red), Mab18-Alexa488 (green) and Mab125-Alexa647 (magenta). Acrosome-reacted spermatozoa were detected with IZUMO1 antibodies (Mab18 and Mab125; shown by arrowheads). To detect the acrosome reaction in IZUMO1-null spermatozoa, Acro-GFP spermatozoa, which have green fluorescence that should disappear from the acrosome, were used to distinguish the acrosome reaction after 2 h of incubation in TYH medium with JUNO-FC and Mab125-Alexa647. Acrosome-reacted spermatozoa are shown by arrowheads. (**d**) IZUMO1-mCherry in spermatozoa showed monomeric brightness. The sperm lysate of IZUMO1-mCherry or COS-7 cell lysates expressing *Myocilin-mChery*, *Cd2-mCherry* or *mCherry* was prepared, and specific brightness of each protein was determined as described in the Methods. The true specific brightness was obtained from the bin time dependence (PCMH, left panel). The ranges of the upper and lower confidence interval (0.95) were also calculated from the five measurements and expressed as error bars of the mean. Brightness of IZUMO1-mCherry was statistically not different from that of mCherry or CD2-mCherry. Error bar represents CI95. (**e**) Immunostaining of acrosome-intact spermatozoa with JUNO-FC. Fresh spermatozoa collected from the epididymis of wild-type mice were mounted on glass slides, dried up and then immunostained with JUNO-FC (red), Mab18-Alexa488 (green) and Mab125-Alexa647 (magenta). Final concentration of all antibodies used was at 0.5 μg ml^−1^. JUNO-FC was added at 1 μg ml^−1^. Nuclei were stained with Hoechst 33342. Scale bar, 20 μm.

Recently, JUNO has been identified as the receptor of IZUMO1 on the egg side^5^. To ascertain the IZUMO1 and JUNO interaction in our system, recombinant JUNO-FC fusion protein (JUNO-FC) was produced in 293T cells. As shown in Fig. 3a, this recombinant at 1 μg ml^−1^ bound selectively to the full-length *Izumo1*-expressing cells and never bound to the DEL5-113, which is consistent with the previous findings^5^. Furthermore, the deletion of Leu25-Leu54 almost entirely abolished the adhesive competency of JUNO-FC (Supplementary Fig. 3a). During this experiment, we noticed that the recombinant JUNO showed no adhesion-inhibitory activity towards the *Izumo1*-expressing cells even at 30 μg ml^−1^ (Supplementary Fig. 3b). Surprisingly, the percentage of JUNO-FC and Mab18-positive-stained cells in the cell–oocyte interface was 0% (10 oocytes and 57 attached cells), whereas 100% of the Mab125-stained cells were positive (Fig. 3b, right graph). The JUNO-FC-binding pattern also resembled that of Mab18, and JUNO-FC was excluded from the interface (Fig. 3b, asterisks; images from different angles are shown in Supplementary Fig. 5c). When the binding of JUNO to live spermatozoa was examined, we found that JUNO-FC bound to spermatozoa only after the acrosome reaction that was IZUMO1-positive and exhibited the same staining pattern as Mab125 as well as Mab18 (Fig. 3c, upper panels, arrowheads). When the IZUMO1-deficient spermatozoa were examined, JUNO-FC lacked binding activity in the spermatozoa both before (Acro-GFP-positive) and after (Acro-GFP-negative, arrowheads) the acrosome reaction (Fig. 3c, lower panels).

From these results, although IZUMO1 apparently oligomerizes in the sperm–egg interface, we reasoned that IZUMO1 remains as a monomer inside the acrosome of spermatozoa, the structure of which is recognized by JUNO-FC. To examine this possibility, we used a single-molecule brightness analysis using previously reported IZUMO1-mCherry transgenic mice^8^ to determine the oligomeric status of intrinsic IZUMO1. This method is based on Poisson statistics and counts the number of photons emitted from the fluorophore to estimate the true number per unit of time from its distribution of certain bin times^15^. As a monomer control, we chose *mCherry* and mouse *Cd2-mCherry*-expressing COS-7 cells because CD2 is a monomeric immunoglobulin superfamily protein of the plasma membrane^16^. In addition, MYOCILIN^17^, which is dimerized through a leucine zipper, was used as a control dimer. This secretory protein also forms larger oligomers; however, their contribution to the PCH was removed by the burst rejection filter as described in the Methods. We solubilized the cells and spermatozoa with Triton X-100, and photon counts were recorded up to 10^6^. Six sets of PCHs were made from bin times of 10–320 μs, and the frequency of photon occurrence was calculated at each bin time after appropriate corrections for various photophysical factors (Fig. 3d, right panel). The obtained bin time-dependent photon occurrence frequency was analysed by a photon-counting multihistogram (PCMH) to estimate the true molecular brightness^18^ (Fig. 3d, left panel). The result clearly indicates that the single-molecule brightness of IZUMO1-mCherry (59.3 kcpms) was statistically no different from mCherry (53.7 kcpms) or CD2-mCherry (58.8 kcpms) and almost half of MYOCILIN-mCherry brightness (133.0 kcpms), clearly indicating that IZUMO1-mCherry is largely monomeric inside the acrosome. This is consistent with BS^3^ crosslink experiments in which an increased dimeric fraction was observed in the intact IZUMO1 in sperm extract; however, the majority of acrosomal IZUMO1 was monomeric as well^12^.

We also measured the single-molecule brightness of the plasma membrane lysates prepared from CD2-mCherry (monomer control)-expressing COS-7 cells, or IZUMO1-mCherry-expressing COS-7 cells by using PCMH. As a result, no significant difference was observed between CD2-mCherry (average: 23.9 kcpms) and IZUMO1-mCherry (average: 23.3 kcpms), proving that IZUMO1 is a monomer on the COS-7 plasma membrane as well (Supplementary Fig. 3c).

When acrosome-intact spermatozoa were stained with IZUMO1 antibodies (such as Mab18 or Mab125) under membrane-permeabilized conditions, IZUMO1 formed a crescent shape in the acrosomal cap region (Fig. 3e). As for recombinant JUNO, it also bound inside the acrosomal cap (Fig. 3e), similar to IZUMO1 antibodies. Together with the PCH results, this indicates that JUNO may be able to recognize monomeric IZUMO1 selectively.

Because the recombinant JUNO does not bind to the COS-7–egg interface and the binding to spermatozoa does require the acrosome reaction, we speculate that IZUMO1 is stored in a dormant, monomeric state in the interior of the acrosome and that after the acrosome reaction, it migrates out towards the plasma membrane of the sperm head where the monomer meets JUNO.

### IZUMO1 no longer binds to JUNO after dimerization

**Figure 4:**
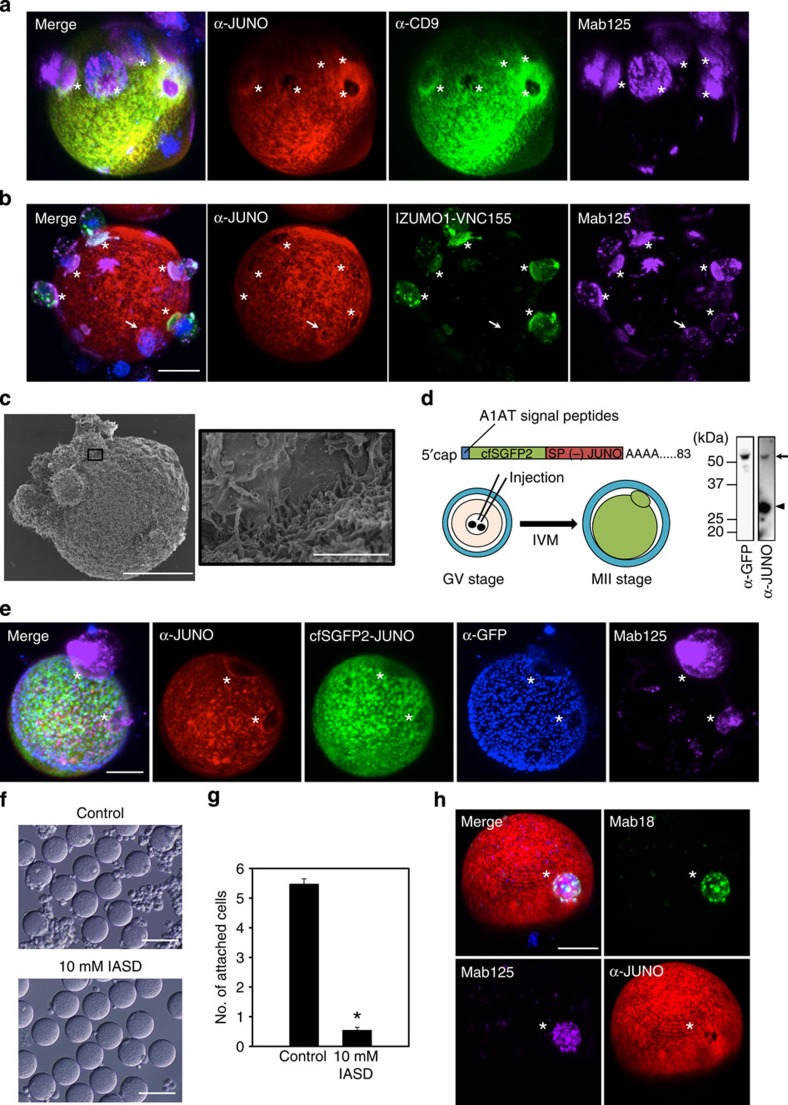
JUNO antibody fails to stain the contact site of an egg and *Izumo1*-expressing cells. (**a**) Distribution of JUNO on oocyte in cell–oocyte assay. Localization of JUNO (TH6, red), CD9 (MZ3, green) and IZUMO1 (Mab125, magenta) in the cell–oocyte assay was observed by each specific antibody. Final concentrations of antibodies were 0.05, 0.5 and 0.5 μg ml^−1^, respectively. Two hours after incubation, images were captured. (**b**) The state of dimer IZUMO1 and JUNO on the egg. In combination with the BiFC analysis, cell–oocyte assays were performed. The asterisks and the arrow indicate adhered cells and the early stage of binding, respectively. Nuclei were stained with Hoechst 33342. Scale bar, 20 μm. (**c**) The scanning electron microscope image of cell–oocyte. Cell–oocyte assays were observed with a scanning electron microscope (left; scale bar, 20 μm). A magnified image of the region surrounded by a black square is shown on the right (scale bar, 2 μm). (**d**,**e**) cfSGFP2-JUNO is excluded from the contact site of the egg and *Izumo1*-expressing cells. Experimental diagram of germinal vesicle (GV) stage oocyte injection. Western blot analysis of the lysate of 15 eggs expressing cfSGFP2-JUNO with α-JUNO (12A5) and α-GFP. Arrow and arrowhead indicate cfSGFP2-JUNO and endogenous JUNO, respectively (**d**). After mRNA microinjection, GV-stage oocytes were incubated in 10% fetal bovine serum/TYH medium for 21 h at 37 °C up to maturation at metaphase II. Then, they were subjected to cell–oocyte assay with Mab125-Alexa546 (magenta), α-GFP polyclonal antibody labelled with α-rabbit IgG antibodies-Alexa405 (blue) and α-JUNO-Alexa647 (red) (**e**). The images were simultaneously taken with autofluorescence (cfSGFP2-JUNO, green). Scale bar, 20 μm. (**f**–**h**) The effects of IASD in a cell–oocyte assay. This membrane-impermeable thiol-reactive reagent drastically decreased the number of attached COS-7 cells expressing *Izumo1*. Cell–oocyte assays were performed with 10 mM IASD. Bright field images were taken 2 h after incubation. Scale bar, 100 μm (**f**), and attached cells were counted (excluding aggregated cells), values are presented as mean±s.e.m., *P<0.0001 (Student's *t*-test; **g**). The oocytes were stained with α-JUNO-Alexa647 (red), Mab18-Alexa488 (green) and Mab125-Alexa546 (magenta) for 30 min after treatment with IASD. Nuclei were stained with Hoechst 33342. Scale bar, 20 μm (**h**).

To confirm IZUMO1 counter-receptor JUNO's function on the egg, we evaluated the effects of antibodies against JUNO or CD9 on the *Izumo1*-expressing COS-7 cells in cell–oocyte assays. As expected, JUNO antibodies selectively inhibited the adhesion of cells (Supplementary Fig. 4a). When localization of JUNO and CD9 on the egg in an *Izumo1*-expressing COS-7-adhered state was examined, we found that antibodies against JUNO (TH6) and CD9 (MZ3) lacked binding affinity to the interface (Fig. 4a) where IZUMO1 formed dimers, as shown by BiFC (Fig. 4b). Interestingly, the signal of Mab125-Alexa546 was often intensified at the edge of the interface where antibodies against JUNO and CD9 were also enriched (Supplementary Fig. 4b). In some cells, we observed the dimeric IZUMO1, as shown by BiFC, which was not observed in the apparent interface, although the edge of the interface was demarcated by the JUNO antibody (Fig. 4b, arrow), suggesting that there may be a ‘touching' stage before complete dimerization. Scrutinizing the interface structure using scanning electron microscopic observation indicated that the COS-7-binding regions of the egg were abundant with microvilli. At the edge of the interface, direct binding of individual microvilli to the flat surface of COS-7 cells was observed (Fig. 4c).

Next, we tested whether the apparent lack of the JUNO antibody at the interface was caused by an inaccessibility of the antibody. As Mab125 can stain the interface, there has to be enough space for diffusion of the antibody; however, it is possible that a particular epitope of monoclonal JUNO antibody was latent in the area. To examine this possibility, we expressed JUNO fused to cfSGFP2, which was developed for imaging in the extracellular environment^14^, by microinjecting its mRNA with a 5′ cap into oocytes at the immature germinal vesicle stage isolated from ovaries (Fig. 4d). The RNA-injected oocytes were cultured for 21 h for *in vitro* maturation. We confirmed cfSGFP2-JUNO expression by western blotting with both α-JUNO- and GFP-specific antibodies (Fig. 4d) and the surface expression of the molecule by the green fluorescent (GFP) antibody without permeabilization (Fig. 4e). As shown in the image of cfSGFP2-JUNO, the GFP signal was nearly absent at the interface, and no accumulation was observed at the rim of the interface as seen by the JUNO antibody (Fig. 4e, asterisks). This result indicates that JUNO was excluded considerably from the adhesive surface (Fig. 4e, asterisks). Because IZUMO1 remained tightly bound at the interface mostly lacking JUNO, we concluded that an unidentified IZUMO1 receptor on the egg side must participate in this event.

We speculated that rapid reduced antigenicity of Mab17 and Mab18 on the surface of the oocyte might be a phenomenon resulting from the disulfide relay system because it is reported that ERp57, one of the major disulfide isomerases, has a crucial role in triggering sperm–egg fusion^19^. Therefore, we used the membrane-impermeable thiol-reactive reagent, 4-Acetamido-4′-((iodoacetyl)amino)Stilbene-2,2′-Disulfonic Acid (IASD), to block any thiol-mediated reaction^20^ and counted the number of attached cells on the oocytes 2 h after incubation in a cell–oocyte assay (Fig. 4f). The number of attached cells markedly decreased to 0.55±0.09 (82 oocytes, *n*=3, mean±s.e.m.) compared with non-treated *Izumo1*-expressing COS-7 cells (5.48±0.17; 60 oocytes, *n*=3, mean±s.e.m.) (Fig. 4g). The adherence of cells with the IASD treatment could be caused by weak nonspecific adhesion, since when immunostaining was performed with Mab18-Alexa488, Mab125-Alexa546 and α-JUNO-Alexa647, the signal of Mab18 and JUNO exclusion remained incomplete, suggesting that they never moved on to the next tight binding step (Fig. 4h).

### JUNO is required to trigger structure conversion of IZUMO1

**Figure 5:**
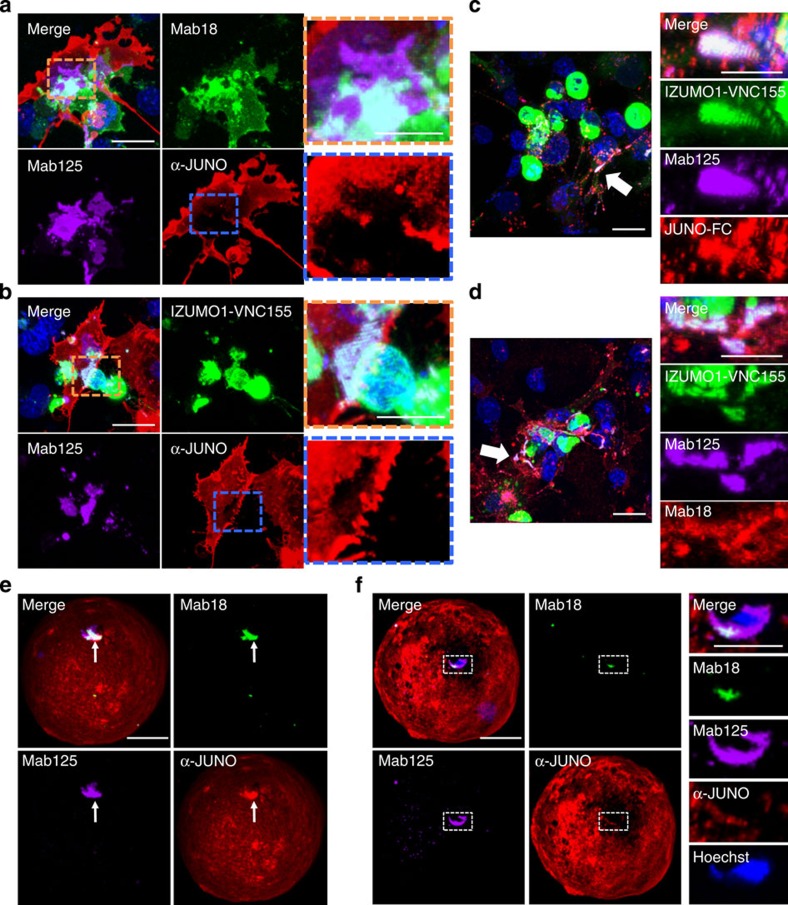
JUNO disappears from the contact site of not only cell–cell but also sperm–egg adhesion. (**a**,**b**) The reconstitution approach in COS-7 cells. COS-7 cells transfected with a mammalian expression vector inserted with *Juno* and *Izumo1* cDNA were mixed and incubated for 24 h before observation using confocal microscopy. Lower magnification images are shown in the left panels. The region of the contact site is shown by a dashed box. (**a**) COS-7 cells were stained with α-JUNO-Alexa647 (red), Mab18-Alexa488 (green) and Mab125-Alexa546 (magenta). (**b**) BiFC analysis (green) was performed with α-JUNO-Alexa647 (red) and Mab125-Alexa546 (magenta). Magnification of the orange or blue dashed box is in the right panels. Nuclei were stained with Hoechst 33342. Scale bar, 20 μm in lower magnification, 10 μm in higher magnification. (**c**,**d**) BiFC in *Izumo1-VNC155*-expressing cells. COS-7 cells were transiently co-transfected with *Izumo1-VN155* and VC155. After 48-h incubation, they were stained with Mab125-Alexa647 (magenta) and JUNO-FC (**c**, red) or Mab18-Alexa546 (**d**, red). After Z-sectioning and 3D reconstitution, magnified images (right panel) were taken from the direction of the arrow. Nuclei were stained with Hoechst 33342. Scale bar, 20 μm in lower magnification, 10 μm in higher magnification. (**e**,**f**) The characteristics of each antibody before and after sperm–egg fusion. Zona-free oocytes and spermatozoa were co-incubated with Mab18-Alexa488 (green), Mab125-Alexa546 (magenta) and JUNO (red) antibodies (**e**,**f**). Sperm–egg fusion was visualized with Hoechst transfer (blue). Images of before (**e**) and after (**f**) fusion were taken after fixation. Highly magnified photographs correspond to the dashed box in the left pictures. Scale bar, 20 μm in lower magnification, 10 μm in higher magnification.

The previous report showed that, after 24 h mixing of *Izumo1*-expressing 293T cells with *Juno*-expressing cells, both IZUMO1 and JUNO seemed to co-exist at the contact site^5^. To confirm this, we did a comparable experiment in COS-7 cells and carefully examined the reconstituted image from the Z-stacks. Intriguingly, we could reproduce the exclusion of JUNO at the interface in cell-to-cell adhesion, consistent with the observation described above (Fig. 5a,b). Moreover, Mab125-Alexa546, but not Mab18-Alexa488, intruded into the contact site (Fig. 5a), and the IZUMO1 was BiFC-positive at the adhesive region (Fig. 5b). However, we did not find any fused cells even after 48 h of co-incubation. As shown in Fig. 2b, BiFC also occurred at the contact site of the cells expressing *Izumo1-VNC155.* During the course of this experiment, we noticed that this dimerization was incomplete or at least distinct from the JUNO-triggered reaction because neither JUNO-FC nor Mab18-Alexa546 were excluded from the contact site of JUNO-negative and IZUMO1-VNC155-positive cells (Fig. 5c,d), indicating that this dimerization essentially differs from the JUNO-triggered dimerization as seen at the contact site (Fig. 5a,b).

Finally, we examined whether the structure conversion of IZUMO1 accompanying JUNO exclusion takes place during the early phase of gamete fusion. To this end, we performed immunostaining with Mab18-Alexa488, Mab125-Alexa546 and α-JUNO-Alexa647 at the very early stage of sperm–egg fusion. Fused spermatozoa were visualized by Hoechst transfer^4^. By scrutinizing the samples, we found that, at the very early phase, JUNO was first enriched at the site of the acrosome-reacted spermatozoa that had attached to the egg plasma membrane (Fig. 5e, arrow). However, at a step after tethering but before completion of fusion, JUNO was lost from the sperm–egg interface that was marked by Mab125 (Fig. 5f). Judging from the shape of the fused spermatozoa in the Hoechst image, they are considered to be in an early phase, within 3 min of fusion (Fig. 5f, Hoechst)^8^. Importantly, even though the equatorial segment, the domain of the sperm acrosome that fuses with the egg membrane, still remained partially reacted with Mab125-Alexa546 (Fig. 5f, Mab125), almost all Mab18-Alexa488 had disappeared from the sperm head (Fig. 5f, Mab18).

## Discussion

The results presented here strongly indicate that monomeric IZUMO1 on the spermatozoon is recruited to the egg by JUNO, and then transferred to an unidentified receptor while forming a dimer, which establishes the tight adhesion of the two cell membranes. To dissect the membrane fusion of spermatozoa and egg, which is an instantaneous process, we have employed a cultured cell–oocyte-binding system that terminates before fusion^12^. Existence of both monomer and dimer forms of IZUMO1 *in vivo* is supported by two lines of evidence: (1) the majority of IZUMO1 in spermatozoa is monomeric, as revealed by single-molecule brightness analysis and (2) complementation of Venus fragments fused to IZUMO1 occurred at the cell–oocyte interface but not on the rest of the cell surface. Previously, we showed that this fragment forms the stable SDS-resistant dimer in solution^12^. To reconcile this with the current results, we have to propose that the monomeric form is converted to dimer on cell–egg interaction and that the structure conversion must accompany the collapse of this N-terminal domain into the interior of the molecule (complex) on oligomerization because both Mab17 and Mab18 bound to Asp5-Leu113 and lost affinity to the contact site of cell–egg. Although this region does not have the typical coiled-coil region similar to that of fusion-related proteins such as SNARE proteins^21^, the structure change as proposed here is reminiscent of highly exergonic assembly of SNARE complexes to overcome the fusion barrier^22^.

What could be the exact mechanism for the proposed rearrangement of the IZUMO1 structure? Different from SNARE, global structures of extracellular proteins are governed by an arrangement of disulfide bonds, and the disulfide bond exchange activity often determines the higher structure. This process is generally completed in the ER; however, it also occurs in the extracellular space. For example, incorporation of lamin into the extracellular matrix requires action of QSOX1 (ref. 23), an enzyme-catalysing disulfide relay^24^. Similarly, ERp57, one of the major disulfide isomerases, was shown to act on a wide spectrum of extracellular matrix proteins^25^. Interestingly, this enzyme is also required for triggering sperm–egg fusion^19^. In fact, we found in this study that the membrane-impermeable thiol-reactive reagent significantly inhibits cell–oocyte binding (Fig. 4f–h). Because the six Cys residues are clustered at the C-terminal flanking region of the Izumo domain among the conserved eight Cys residues and their rearrangements could exert the global structure transition, regulation of the disulfide bond may be involved in the activation of the fusogenic process after JUNO–IZUMO1 interaction.

**Figure 6:**
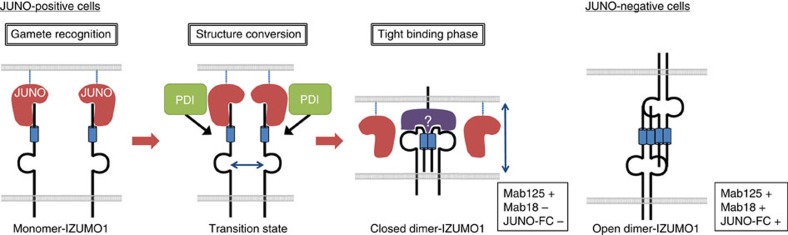
Schematic model for the IZUMO1-JUNO interaction. In gamete recognition, JUNO first binds to monomeric IZUMO1. By the weak, but specific interaction, an increased amount of JUNO gradually gathers to the attached site of the spermatozoon, which also increases the chance of IZUMO1 self-collision, to induce its dimerization (open dimer form) by depletion association. By the action of PDI-related protein, in the tight binding phase, IZUMO1 bends the entire structure towards the sperm membrane side by a thiol-disulfide exchange reaction. In particular, the N terminal of IZUMO1 (Asp5-Leu113) is folded on to the interior side of the molecule to form a closed dimer. The molecule no longer binds to JUNO and instead binds to a putative oocyte receptor. Presumably, the free energy change by the binding could be close to the level to overcome the charge repulsion of the two phospholipid bilayers. On the other hand, in the case of cells without JUNO, dimeric IZUMO1 could be formed by the presence of IZUMO1 in the juxtaposing membrane probably by the principle of exclusion volume-induced self-association of IZUMO1. However, this structure conversion is incomplete unlike the closed form, which is triggered by JUNO, so that Mab18 or JUNO-FC remains able to bind.

This study also suggests that JUNO interaction constitutes the early phase of the IZUMO1-mediated process. This hypothesis is based on the current two observations: apparent lack of JUNO antibody reactivity to the attachment area and the excluded localization of cfSGFP2-JUNO at the cell–egg interface (Fig. 4e). The affinity of JUNO to IZUMO1 was reportedly very low (*K*_d_=12.3 μM), and a pentamerized IZUMO1 ectodomain construct, which includes the cartilage oligomeric matrix protein that forms pentamers to enhance the affinity of the IZUMO1–JUNO interaction, was required to uncover the specific JUNO–IZUMO1 interaction^5^. Assuming that the IZUMO1 fragment in the pentamerized IZUMO1 construct contains the molecule in a dormant state, it is likely that JUNO plays a role in the initial gamete recognition and enhances the surface density of IZUMO1 at the contact site. This could probably facilitate structure conversion through the principle of excluded volume depletion^26^. Several examples have been reported showing that molecular crowding could cause global structure change^27^. While development of a probe to selectively label the dimeric IZUMO1 and reconstituting the exact structure would be needed to directly prove this hypothesis, all of our current results are consistent with the following proposed model: Step1, in gamete recognition, since IZUMO1 is in a monomeric form in mature spermatozoa, JUNO selectively recognizes the monomeric IZUMO1. Step2, after IZUMO1–JUNO recognition, IZUMO1 is clustered by JUNO, accompanied by the transient dimerization, presumably through the effects of depletion attraction. Step3, in the tight binding phase, by the action of PDI-related protein, the overall structure of IZUMO1 is reconstructed so that the N terminus of IZUMO1 (Asp5-Leu113) is folded on to the interior side of the molecule. The closed dimer then binds to the putative oocyte receptor, bringing the juxtaposing phospholipid bilayers into the closest proximity. This process can be probed by the loss of reactivity to Mab18 and JUNO (Fig. 6).

This study also indicates the presence of another factor(s) directly tethering cells to an egg through IZUMO1 as a post-JUNO-binding step. Involvement of CD9 in the process has been studied extensively. Recently, by using a one-to-one dual pipette assay, Chalbi *et al.*^28^ showed that *Izumo1*-expressing K562 cells have the potential to bind to the egg plasma membrane. Initiation of this strong cell–egg adhesion was observed within ∼5 min of contact. However, because recruitment of CD9 to the contact area was followed by IZUMO1 (ref. 28) and CD9-null eggs essentially maintained the binding capability of IZUMO1 (ref. 12), CD9 is unlikely the putative tethering factor. Still, the importance of CD9 is clear in the sperm–egg interaction, as sperm–egg adhesive strength was reduced by its gene disruption^29^. This could be a result of the impaired development of microvilli of eggs that CD9-deficient mice showed in a previous study^30^. Cell–egg adhesion contact was indeed observed mostly in an area where microvilli were highly enriched (Fig. 4c). Given that JUNO exclusion occurs even at the surface of COS-7 cells expressing *Izumo1* and *Juno* (Fig. 5a,b), the unidentified oocyte receptor may be a general cell adhesion molecular such as integrin. However, we are not excluding the possibility that the factor the closed dimer recognizes may be a non-proteinous factor such as a phospholipid.

Recently, it was found that glycosylphosphatidylinositol (GPI)-anchored TEX101 protein is released from mature spermatozoa by angiotensin-converting enzyme utilizing its GPIase activity so that the spermatozoa acquires fertilizing ability^31^. JUNO also contains a GPI anchor and becomes undetectable from the surface 40 min after fertilization^5^. Perhaps, our finding that JUNO was nearly absent at the interface of both cell–oocyte and sperm–egg may relate to this phenomenon. In addition, this phenomenon occurs even in COS-7 cells expressing *Izumo1* and *Juno* (Fig. 5a,b). From these observations, common GPIase may regulate the JUNO density of the contact site by their GPIase activity, presumably for proceeding to the next step.

Furthermore, the cell-oocyte system is indeed heterologous, so it may terminate before fusion. Conversely, it can be said that spermatozoa are equipped with complete fusion machinery that does not exist other than in spermatozoon. On the sperm side, only two factors, IZUMO1 (ref. 4) and SPACA6 (ref. 6), actively participate in gamete fusion. As this has just been unveiled, the detailed molecular mechanism will need to be resolved.

In conclusion, based on the results of various approaches, we propose that when an egg meets a spermatozoon, monomeric IZUMO1 is first recognized by JUNO on the egg, quickly dimerizes and JUNO is then replaced with an unidentified receptor, whose interaction may force the juxtaposing membranes in close proximity to repress their repulsion.

## Methods

### Animal

All animal experiments were approved by the Animal Care and Use Committee of Fukushima Medical University, Japan. IZUMO1 knockout^4^, IZUMO1-mCherry transgenic^8^ and Acro-GFP^32^ mice were kindly provided from Osaka University.

### Preparation of antibodies

For Mab17 and 18, female rats were immunized with a KLH-conjugated IZUMO1_5–113_ peptide fragment, and spleen cells were subjected to cell fusion with P3U1 cells. Enzyme-linked immunosorbent assay as the first screening was used. Hybridomas expressing anti-IZUMO1 antibodies were then further selected using indirect immunofluorescence staining and western blot analysis using a hybridoma culture as a source of primary antibodies. Each hybridoma culture was compared in terms of reactivity against proteins with a molecular weight of 56 kDa in the sperm extract. OBF13 and Mab125 were kindly gifted by Dr Okabe (Osaka University). Fluorescein isothiocyanate (FITC) -conjugated α-mouse CD9 monoclonal (MZ3), Alexa Fluor 647–labelled α-mouse FR4 (JUNO) monoclonal (TH6) and unlabelled α-mouse FR4 (12A5) antibodies were purchased from BioLegend. α-GFP polyclonal antibody was produced in our laboratory^14^. Mab17, Mab18, Mab125, TH6 and MZ3 were produced by rats, whereas recombinant JUNO-FC and α-GFP polyclonal antibodies have affinity to α-mouse IgG and α-rabbit IgG, respectively. We used species-specific secondary antibodies to avoid crossreactivity.

### *In vitro* fertilization

Mouse spermatozoa were collected from the cauda epididymis and were capacitated *in vitro* for 2 h in a 200-μl drop of TYH medium that was covered with paraffin oil. B6D2F1 female mice (>8-week old) were superovulated with an injection of 7.5 IU of human chorionic gonadotropin (hCG) 48 h after a 7.5-IU injection of equine chorionic gonadotropin (eCG). The eggs were collected from the oviduct 16 h after the hCG injection. Eggs were placed in a 200-μl drop of the TYH medium. These eggs were incubated with 2 × 10^5^ spermatozoa ml^−1^ for 2 h at 37 °C in 5% CO_2_ with α-IZUMO1 monoclonal antibodies, and unbound spermatozoa were washed away. Eggs were observed 24 h after insemination for the two-cell development under a Hoffman modulation contrast microscope.

### Preparation of IZUMO1 fragments

DNA coding for the extracellular region of mouse IZUMO1 was constructed using synthetic DNA with codon use that was optimized for expression in *Escherichia coli*. IZUMO1 fragments were amplified using PCR with a 3′-primer with sequence encoding Ala-Gly-Gly-His-His-His-His-His-His, a linker plus a hexahistidine tag. Amplified PCR products were cloned back into pAED4. IZUMO1_5–113-Pro_ was constructed using the PCR-based mutagenesis to introduce prolines at the positions of Leu70, Leu81, Leu94 and Leu104 of IZUMO1_5–113_ without a linker and a hexahistidine tag. All constructs were expressed using *E. coli* strain BL21 (DE3) pLysS (Agilent Technologies) or Rosetta (Merck Millipore). IZUMO1_5–113_, IZUMO1_5–113-Pro_ and IZUMO1_Ig domain_ were strongly expressed and accumulated in inclusion bodies. Inclusion bodies from 1.6 l of culture medium were suspended in 3 ml of 10 mM Tris-HCl (pH 8.5) and were solubilized by the addition of 3 g of solid guanidine hydrochloride (GdnHCl). These samples were then purified over a Superdex 75 (GE Healthcare) that was pre-equilibrated with 6 M urea in 10 mM Tris-HCl (pH 8.5). On the other hand, IZUMO1_5–56_, IZUMO1_57–113_, IZUMO1_26–113_ and IZUMO1_45–113_ were recovered in the soluble fraction and were purified using a HiTrap metal-chelating column (GE Healthcare). Finally, all samples were purified using reverse-phase chromatography. MALDI-TOF mass spectrometry confirmed that the molecular weights of purified IZUMO1 fragments were identical to the expected values that were calculated from their amino-acid sequences (with an error of ±0.025%). Protein stock concentrations were determined by measuring the absorbance at 280 nm in 6 M GdnHCl and 20 mM sodium phosphate (pH 6.5). Molar extinction coefficients were calculated based on the number of Trp and Tyr residues using the Edelhoch spectral parameters.

### Measurements of SPR spectra

The antibodies were immobilized to the CM-5 sensor chip by the amine-coupling protocol, supplied by the manufacturer, at 25 °C using Biacore 2000 (GE Healthcare). The SPR spectra of the IZUMO1 fragments were measured in 10 mM HEPES, 150 mM NaCl, 3 mM EDTA and 0.005% Tween-20 at 20 °C, and the bound fragments were washed with 10 mM glycine and 0.5 M NaCl at pH 2.0. All experiments were duplicated.

### Cell–oocyte assay

The cloned mouse *Izumo1* cDNA was ligated into the mammalian expression vector pCXN-2, and COS-7 or 293T cells were transiently transfected with this vector using polyethylenimine methods. After 2 days, transfected COS-7 or 293T cells were collected with 10 mM EDTA-PBS, washed three times with PBS and suspended in TYH medium (LSI Medience). For preparation of zona-free eggs, B6D2F1 female mice (>8-week old) were superovulated with an injection of 7.5 IU of hCG 48 h after a 7.5-IU injection of eCG. The eggs were collected from the oviduct 16 h after the hCG injection. Eggs were placed in a 200-μl drop of the TYH medium. The zona pellucida was removed from eggs by treating with 1.0 mg ml^−1^ of collagenase (Wako). Zona-free eggs were incubated with transfected COS-7 or 293T cells at 37 °C in the TYH medium for 2 or 1 h, respectively. IZUMO1 was stained by anti-IZUMO1 monoclonal antibodies fluoresceinated with Alexa Fluor 488, 546 or 647. Eggs were stained with FITC-conjugated α-mouse CD9 (MZ3; BioLegend) and Alexa Fluor 647-labelled α-mouse JUNO (TH6; BioLegend). JUNO-FC and α-GFP were detected using Alexa Fluor 546-conjugated goat α-mouse IgG (H+L), highly cross-adsorbed, and Alexa Fluor 405-conjugated goat α-rabbit IgG (H+L), respectively (Life Technologies).

### Fluorescence imaging

Cell–egg complexes were prepared as described above. Complexes were incubated with the appropriate antibodies with 1 μg ml^−1^ of Hoechst 33342 for 1 h at 37 °C in the TYH medium. After the eggs had been washed several times in the TYH medium by transferring spots of oocytes containing media, the eggs were observed. A × 100 oil-immersion objective (numeric aperture 1.49) was used to capture confocal images with an A1R microscope (Nikon). Pinhole was set at 3.0 airy unit. For three-dimensional (3D) reconstruction, 100–120 fluorescent images were taken at 1-μm (for oocytes) or 0.2-μm (for cultured cells in Fig. 5a–d) intervals on the *z* axis, and then 3D images were reconstructed using the built-in software NIS-Elements ver. 4 (Nikon).

### BiFC construction

We employed a Venus V150A point mutation in this assay because this mutant increased the signal-to-noise ratio by 8.6-fold^33^. For construction of the IZUMO1 fusion protein, the cDNA fragment encoding mouse *Izumo1* was amplified using the primer set 5′-GCTCTAGAGCGCCGCCATGGGGCCGCATTTTACACT-3′/5′-GGCTCGAGGTTTTCTGTTGCCTCGCTCTTATCT-3′ and was ligated into the *Xba*I/*Xho*I site of the pCXN2 vector. To construct IZUMO1-VN155 and IZUMO1-VC155, we used *mVenus* cDNA as a template, and cDNA fragments and linkers for VN155 and VC155 were amplified using PCR with primer sets 5′-GGCTCGAGCGCTCCATCGCCACGATGGTGAGCAAGGGCGAGGAGCTGT-3′/5′-CCGAATTCTTAGGCGGTGATATAGGCGTTGTGGCT-3′ (linker is RSIAT; with V150A point mutation) and 5′-GGCTCGAGCGCCCGGCCTGCAAGATCCCGAACGACCTGAAACAGAAGGTCATGAACCACGACAAGCAGAAGAACGGCATCAAGGCCAAC-3′/5′-CCGAATTCTTACTTGTACAGCTCGTCCATGCCGAG-3′ (linker is RPACKIPNDLKQKVMNH), respectively. The amplified cDNA fragments were cloned into the above pCXN2/IZUMO1 vector digested with *Xho*I/*EcoR*I. IZUMO1 deletion mutants were generated by the invert PCR of KOD-Plus-neo Mutagenesis (Toyobo) using primer set 5′-CAGGTGGTCATTCAGGTAAGTGTTT-3′/5′-GGGGCCGTGGACGAGAACACACTGG-3′ for the 25-54 deletion mutant and 5′-ACATTTGATGCAGGGCCTCCCTGGA-3′/5′-TGTCCCAACAAATGCGGAGTGATGT-3′ for the 5–113 deletion mutant). All constructs were confirmed using DNA sequencing.

### Fluorescence recovery after photobleaching analysis

Fluorescence recovery after photobleaching analysis of COS-7 cells expressing either IZUMO1-Venus or VNC155 was carried out essentially as described^34^. A spot (2.5 μm) in the plasma membrane or the ER-like intracellular structure was photobleached with a 488-nm laser of the Nikon A1R confocal microscope through a × 60 water-immersion objective lens (numeric aperture 1.27) at 0.026 mW and then the recovery of the fluorescence was monitored. The fluorescence intensity of the 10 frame immediately before the photobleaching was averaged and used to normalize the intensity during the recovery. Recovery profiles of the normalized intensity *F(t)* in the bleached area at time *t* were fit to a closed form solution^35^ of the diffusion equation using modified Bessel function,


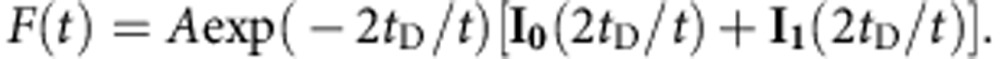


**I**_**o**_ and **I**_**1**_ are modified Bessel functions, *A* and *τ*_D_ are the maximum recovery rate and the diffusion time, respectively. Best fit values were obtained using Excel 2013 and Origin2015 (OriginLab).

### Purification of JUNO-FC fusion protein

The mouse FR4 (JUNO; residues 20–221) cDNA excluding the secretion signal peptide (residues 1–19) and glycophosphatidylinositol anchor signal peptide (residue 222–244) were expressed fusing with the CH2 and CH3 domains of the mouse IgG heavy chain and the hinge region from the expression vector pCMV6-AC-FC-S (OriGene). This construct uses a human interleukin-2 leader sequence at the N terminus. For expression, 293T cells were transiently transfected with the JUNO-FC DNA using the polyethylenimine methods^36^. These cells were maintained in 1,000 ml of DMEM without phenol red supplemented with 2% fetal bovine serum, 1 mM sodium pyruvate, 1 × non-essential amino acids (Life Technologies), 2 mM L-Glutamine, 1,000 units of penicillin and 1 mg of streptomycin at 37 °C. The medium was collected after 4 days and debris filtrated using a 0.45-μm filter before loading on HiTrap rProtein A FF columns (GE Healthcare). The column was washed with 20 mM phosphate buffer (pH 7.0) and eluted with 0.1 M sodium citrate (pH 5.0) and neutralized with 1.0 Tris-HCl (pH 9.0). Peak fractions were pooled and finally dialysed against PBS at 4 °C. The protein purity was judged using SDS–PAGE.

### Plasma membrane preparation

The plasma membrane sheet fraction was prepared from COS-7 cells essentially according to a method by Tuma and Hubbard^37^ with a modification for subcellular fractionation of cultured cells^38^. After removing the culturing medium, the cells were scraped into 0.5 ml of ice-cold homogenizing buffer (0.25 M sucrose/1 mM MgCl_2_/10 mM Tris-HCl, pH 7.4) and homogenized by repeated suction 10 times using a 250-μl Hamilton microsyringe. All procedures were carried out at 4 °C. The homogenates were centrifuged at 250 *g* for 5 min and the supernatant was further spun at 1,500*g* for 10 min. The pellet, the crude plasma membrane fraction, was directly re-suspended in 200 μl of 1.42 M sucrose solution containing 1 mM MgCl_2_ and 10 mM Tris-HCl, pH 7.4 using a 200 μl pipettor five times, and diluted with 3 ml of the same 1.42 M sucrose solution. The membrane suspension was transferred into a tube for a RPS52 rotor, overlaid with 1.25 ml of the homogenizing buffer and centrifuged for 1 h at 113,000 *g* without a break in a Hitachi CS 100GXL. The interface of 0.25 M sucrose and 1.42 M sucrose was collected and mixed with an equal volume of the homogenizing buffer. The fraction was centrifuged at 10,000*g* for 10 min and the pellet was solubilized with 50 μl of 1% Triton X-100 containing PBS for the PCMH measurement.

### Single-molecule brightness analysis

Molecular brightness was measured essentially as described^14^. Cell lysates were prepared from COS-7 cells expressing *mCherry* or *mCherry-fused human Myociln* or *mouse Cd2* at 1 day post transfection or from IZUMO1-mCherry from spermatozoa^8^. They were solubilized with 1% Triton X-100/PBS with protease inhibitor cocktail (Wako), and the supernatants after a 30-min spin at 15,000*g* were used for analysis. Each lysate was diluted with the solubilization buffer to obtain 20,000–30,000 counts per molecule per second and placed on a coverslip. The specific brightness of each mCherry fusion protein obtained under the same condition was determined.

### Determination of specific brightness

The fluorophore was excited by a 561-nm diode laser of A1R, and the light was collected through LP590 filter (Nikon) with a × 60 water-immersion objective (numeric aperture 1.27). The signal was fed to a single-photon counting avalanche photodiode (PicoQuant) of PicoHarp300 (PicoQuant) operated by Symphotime 64. PCHs were generated from a single measurement (1–1.5 × 10^6^ counts) with a bin time of 0.01, 0.02, 0.04, 0.08, 0.16 and 0.32 ms. If we consider the volume, *V*_0_, to be large enough to contain all photons, the probability of detecting *k* photon in a volume *V*_0_ from one molecule is expressed as


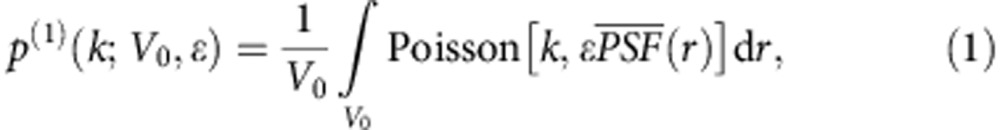


where *ɛ* is the molecular brightness and 
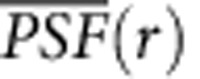
 is the scaled point spread function (PSF). The volume of PSF, *V*_PSF_, is


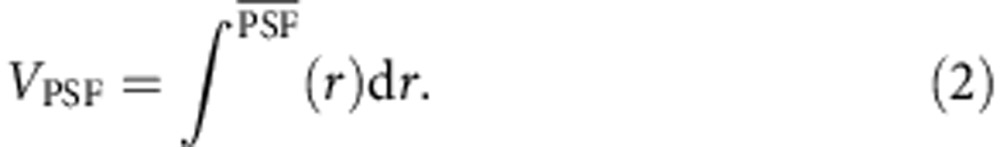


The average of *k* photons is the product of the brightness parameter, *ɛ* and the ratio *V*_PSF_/*V*_0_. *V*_0_ was set proportional to the observation volume. In the actual measurements, total PCH containing *N* molecules becomes the *N*th convolution of (1):


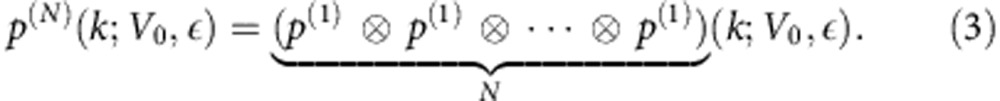


For correction of an out-of-focus photon, which largely deviates PCH from a 3D Gaussian profile, second-order correction was applied to consider two photons from the out-of-focus region, as previously described^18^,^39^. Dynamic (3D diffusion) and photochemical (triplet) processes were also corrected, and the best-fit values of *ɛ* were obtained from each PCH at a bin time. As predicted by the theory of PCMH^40^, *ɛ* at each bin time showed significant time dependence, which was caused by the widening of the histogram by various processes at longer bin times. The specific brightness of the molecule was therefore determined from the time-dependent decay according the PCMH model. All PCH calculations were made using FFS Data Processor ver. 2.3 (SSTC). To avoid disturbance of the Poisson process due to aberrant photon bursts, we used a burst rejection function of the software.

### Scanning electron microscope

Cell–egg complexes were prepared as described in Methods and were placed on RetroNectin (Takara Bio)-coated cover glass for 30 min at 37 °C. Primary fixation was performed in 4% paraformaldehyde, 2.5% glutaraldehyde, 2 mM CaCl_2_ and 0.5% polyvinylpyrrolidone in HEPES buffer (pH 7.0). After fixation overnight at 4 °C, these samples were washed with PBS three times and transferred into 2% osmium tetroxide in PBS at 4 °C for 1 h. The samples were dehydrated through a graded series of ethanol and replaced with *tert*-Butylalcohol. The samples were dried in a *tert*-Butylalcohol freeze-drying apparatus (VFD-21S, Shinku Device) and were transferred on a stub with silver paste plus thinner and coated with osmium in an HPC-1SW coater (Shinku Device). An SU8220 scanning electron microscope (Hitachi) was used for imaging according to the manufacturer's protocol.

### *In vitro* transcription

Mouse *Juno* cDNA was amplified by PCR using primer sets 5′-CCTGTACAAGGGGGACAAACTGCTCAGCGTCTGCA-3′/5′-CCGCGGCCGCTCAGGGATGGAACAACAGGCACAGA-3′ and subcloned into the *Bsr*GI/*Not*I sites in ss-cfSGFP2 vector^14^. This construct consists of human α1-antitrypsin (A1AT1) signal sequence, cfSGFP2, and mouse JUNO (residues 20–221; Fig. 4d). To insert this DNA fragment into the vector for mRNA synthesis (pcDNA3.1 that contained poly(A) repeats comprising 83 adenines to extend poly(A) tail, kindly provided by Dr Yamagata at Kinki University), the DNA fragment was amplified by PCR using primer sets 5′-GGCAATTGGCCGCCATGCCGTCTTCTGTCTCGTGG-3′/5′-GGAAGCTTGTACCATGGGGGTG-3′ and ligated into *Eco*RI/*Not*I sites in the above vector. The cDNA-containing vectors were linearized with *Xba*I and used as templates for RNA synthesis using the RibMAX Large Scale RNA production system-T7 (Promega) with Ribom^7^G Cap Analog (Promega). After the synthesized RNAs had been qualified with agarose gel electrophoresis and quantified using ultraviolet absorbance using a spectrophotometer, the RNA precipitates were dissolved in RNase/DNase-free water. For injection, the final concentration of RNA was adjusted to 300 ng μl^−1^.

### Microinjection of mRNA

For microinjection, immature oocytes were isolated from the ovaries of B6D2F1 female mice (>8-week old) 46 h after injection of eCG (7.5 units). Immature oocytes (germinal vesicle stage) were collected from ovaries using very fine forceps in FHM medium with 0.01% (w/v) hyaluronidase covered with mineral oil (Sigma-Aldrich) at room temperature. The released oocytes were collected with glass capillaries and placed in a 20-μl drop of FHM medium. Then, the synthesized RNA was injected into the nucleus of the oocytes with glass capillaries attached to a micromanipulator (FemtoJet, Eppendorf). The injection volume was ∼5 pl per oocyte, which corresponded to 1.5 pg mRNA. After RNA injection, the oocytes were incubated in 10% fetal bovine serum/TYH medium for 21 h at 37 °C up to maturation at metaphase II. The oocytes were then subjected to zona pellucida removal by brief incubation in acid Tyrode's solution (Sigma-Aldrich) and were subjected to a cell–oocyte assay.

### Sperm–egg fusion assay

Zona-free oocytes were prepared as stated above. They were preloaded with 1 μg ml^−1^ Hoechst 33342 (Sigma) in TYH medium for 10 min and were washed before the addition of the spermatozoon. Zona-free eggs were co-incubated with 2 × 10^5^ mouse spermatozoa ml^−1^ for 30 min at 37 °C in 5% CO_2_ with Mab18-Alexa488, Mab125-Alexa546 and α-JUNO-Alexa647 antibodies. After 30 min of incubation, the eggs were observed under a confocal fluorescence microscope after fixing with 4% paraformaldehyde. This procedure enabled the staining of only fused sperm nuclei by transferring the dye into spermatozoa after membrane fusion.

## Additional information

**How to cite this article:** Inoue, N. *et al.* Oocyte-triggered dimerization of sperm IZUMO1 promotes sperm–egg fusion in mice. *Nat. Commun.* 6:8858 doi: 10.1038/ncomms9858 (2015).

## Supplementary Material

Supplementary Information Supplementary Figures 1-5
