# Supplementary material for: Oocyte-triggered dimerization of sperm IZUMO1 promotes sperm–egg fusion in mice
Source: Nat Commun. 2015 Nov 16;6:8858. doi: 10.1038/ncomms9858 (PMC4660202; doi:10.1038/ncomms9858)
Supplement: Supplementary Information — Supplementary Figures 1-5 [file ncomms9858-s1.pdf]

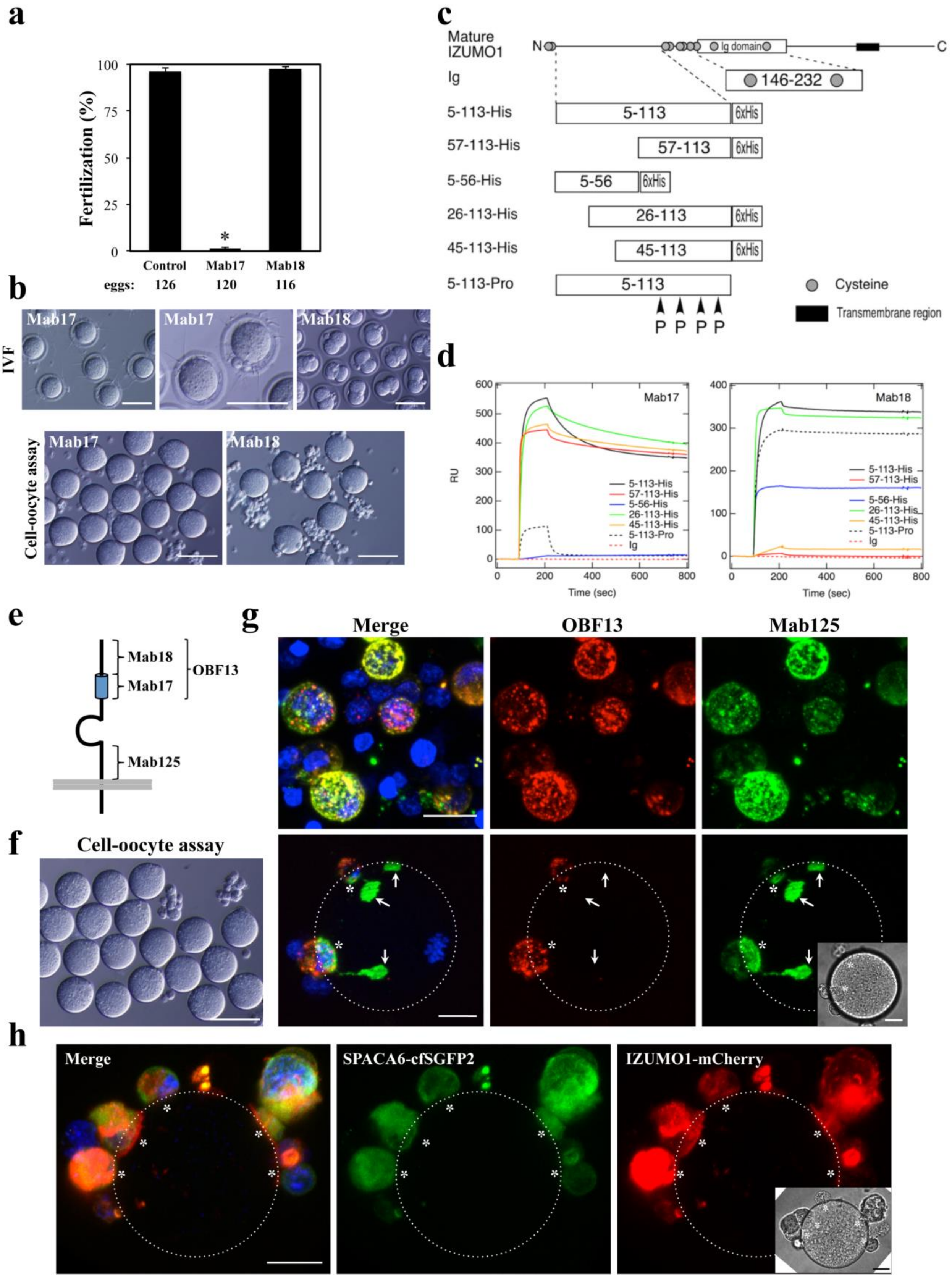
a
Fertilization (%)
Control
Mab17
Mab18
eggs: 126 120 116
b
IVF
Mab17
Mab18
Cell-oocyte assay
c
Mature IZUMO1
N
C
Ig domain
Ig
146-232
5-113-His
57-113-His
5-56-His
26-113-His
45-113-His
5-113-Pro
6xHis
Cysteine
Transmembrane region
d
RU
Time (sec)
e
Mab18
Mab17
OBF13
Mab125
f
Cell-oocyte assay
g
Merge
OBF13
Mab125
h
Merge
SPACA6-cfSGFP2
IZUMO1-mCherry

Supplementary Figure 1. Properties of various IZUMO1 monoclonal antibodies and behavior of SPACA6.

(a) The inhibitory effects of new antibodies (Mab17 and Mab18). They were investigated in *in vitro* fertilization (IVF). The IVF rate was assessed after two-cell development. The values are presented as mean ± s.e.m, (n=3), *p-value was less than 0.0001 (between control or Mab18 and Mab17 treatment) (student's *t*-test).

(b) Representative photos of IVF and cell-oocyte assays. At 24 h after insemination with antibodies, the images were captured (top). The top middle panel is a two-fold magnified image of the left panel (Mab17 treatment), showing that many spermatozoa accumulated in the perivitelline space. The inhibitory effects on cell-oocyte assays were also tested (bottom). Scale bars, 100 μm.

(c) Mature mouse IZUMO1 and its fragments used in surface plasmon resonance (SPR) analyses of antibodies.

(d) SPR spectra of Mab17 (left) and Mab18 (right). 5-113-His, 57-113-His, 5-56-His, 26-113-His, 45-113-His, and 5-113 Pro, which has four Pro mutations to break the helical and Ig fragments of IZUMO1, are shown in black, red, blue, green, orange, and black-and-red dashed lines, respectively. As shown in the micrograph, Mab17 has affinity to 5-113-His, 57-113-His, 26-113-His, and 45-113-His, whereas Mab18 has affinity to 5-113-His, 5-56-His, 26-113-His, and 5-113 Pro.

(e) A schematic diagram of the binding sites of IZUMO1 monoclonal antibodies in this study.

(f) Transmission image of a cell-oocyte assay with incubation of OBF13 at 10 μg/ml. Binding to the egg was completely inhibited at this concentration. Scale bars, 100 μm.

(g) Properties of OBF13 antibody**.** The OBF13 signal showed punctuated localization on the cell surface, same as Mab125, but had a different staining pattern from Mab125 as OBF13 was nearly absent at the egg-cell interface. In the *Izumo1*-expressing COS-7-oocyte assay, OBF13-Alexa546 was used at 0.2 μg/ml (red) and Mab125-Alexa488 at 0.5 μg/ml (green). Both asterisks and arrows indicate the adhesive area. The arrows further indicate the COS-7 plasma membrane which remained bound to the egg. Blue, nuclei stained by Hoechst 33342. Inset shows middle DIC image among Z-stacks. Scale bars, 20 μm. The image of the oocyte is marked by dotted lines.

(h) SPACA6 has not accumulated at the interface of cell–oocyte. COS-7 cells were co-transfected with *Izumo1-mCherry* and *Spaca6-cfSGFP2* cDNAs. After these cells were incubated with oocytes for 2 h, the 3D image was reconstituted from the z-sectioned optical slices. Asterisks indicate the contact area. Nuclei were stained by Hoechst 33342. Inset shows middle DIC image among Z-stacks. Scale bar, 20 μm. The image of the oocyte is marked by dotted lines.

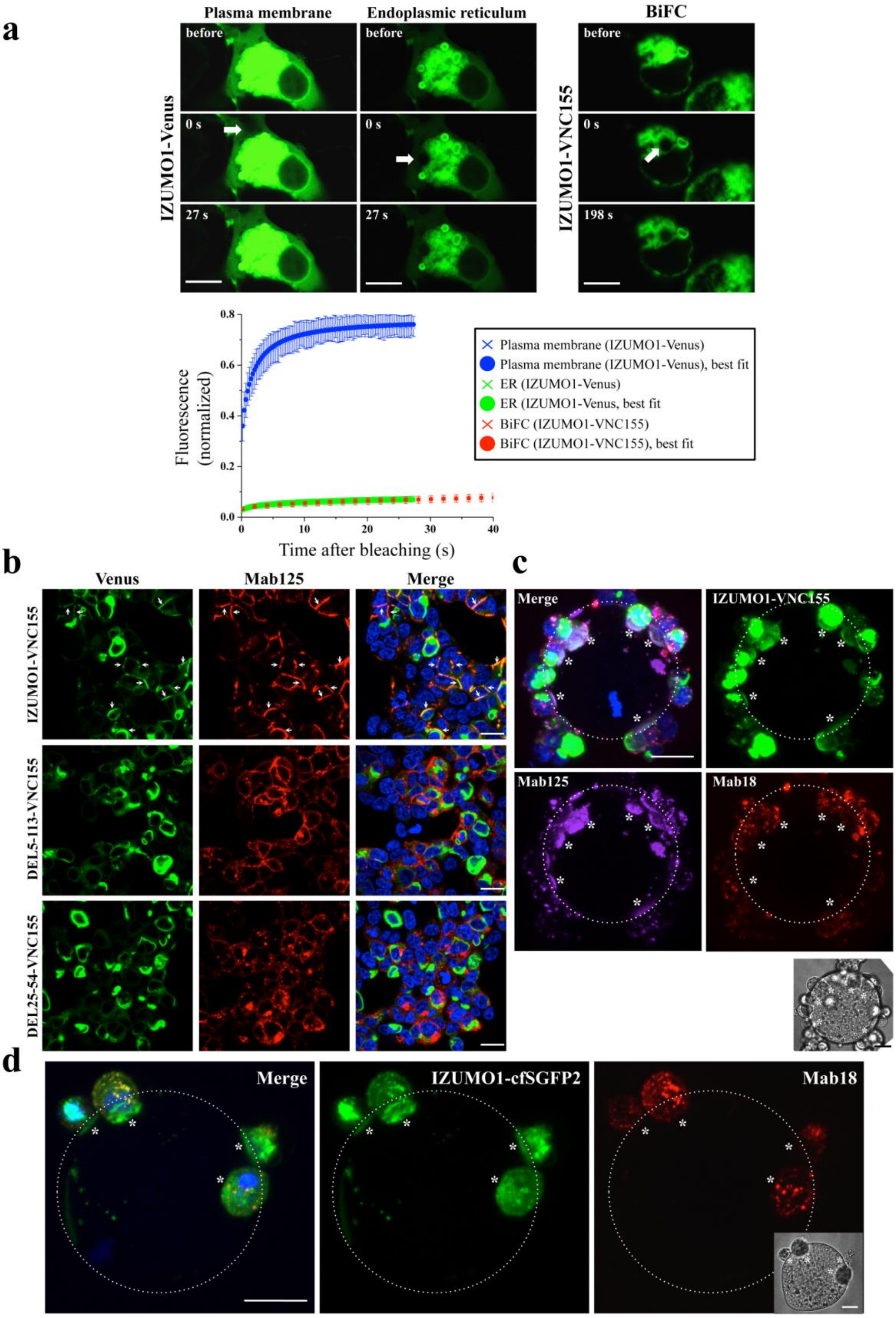
a
Plasma membrane
Endoplasmic reticulum
BiFC
IZUMO1-Venus
IZUMO1-VNC155
before
0 s
27 s
198 s
Fluorescence (normalized)
Time after bleaching (s)
Plasma membrane (IZUMO1-Venus)
Plasma membrane (IZUMO1-Venus), best fit
ER (IZUMO1-Venus)
ER (IZUMO1-Venus, best fit
BiFC (IZUMO1-VNC155)
BiFC (IZUMO1-VNC155), best fit
b
Venus
Mab125
Merge
IZUMO1-VNC155
DEL5-113-VNC155
DEL25-54-VNC155
c
Merge
IZUMO1-VNC155
Mab125
Mab18
d
Merge
IZUMO1-cfSGFP2
Mab18

Supplementary Figure 2. A property of IZUMO1 in COS-7 cells, a series of BiFC assays in 293T cells and localization of IZUMO1-cfSGFP2 in cell-oocyte assay.

(a) The majority of transiently expressed *Izumo1* in COS-7 cells was aggregated and remained intracellular. COS-7 cells expressing *Izumo1-Venus* and *Izumo1-VNC155* were photobleached as described in Materials and methods. Images of a whole cell before and after photobleaching are shown (top). The white arrow indicates a photobleached spot. Fluorescence recovery after photobleaching (FRAP) in IZUMO1-Venus and IZUMO1-VNC155 was observed until 27 and 198 sec after photobleaching, respectively. Error bar represents CI95. Scale bars, 10 μm.

(b) BiFC analysis in 293T cells. BiFC detected at the surface of 293T cells expressing *Izumo1* in addition to intracellular structures. 293T cells were co-transfected with either full-length IZUMO1, DEL5-113, or DEL5-54-truncated IZUMO1, each of which was fused to both VN155 and VC155 (IZUMO1-VNC, green). Before observation, the live cells were stained with Mab125-Alexa546 (red) at 0.5 μg/ml. Although the complementation signal was observed in intracellular structures in all experiments, only the full-length IZUMO1 rendered the plasma membrane signal (arrow), which was marked by Mab125-Alexa546. Scale bars, 20 μm.

(c) BiFC analysis in a cell-oocyte assay in 293T cells. In combination with the BiFC analysis, cell-oocyte assays were performed. The asterisks indicate adhered cells. IZUMO1 was stained with both Mab18-Alexa546 (red) at 0.5 μg/ml and Mab125-Alexa647 (magenta) at 0.5 μg/ml, and a green fluorescence image derived from BiFC (top) or Venus (bottom) was captured simultaneously (green). The adhesive areas were clearly seen and overlapped with BiFC and Mab125-Alexa647 staining. Nuclei were stained by Hoechst 33342. Inset shows middle DIC image among Z-stacks. Scale bars, 20 μm. The image of the oocyte is marked by dotted lines.

(d) Localization of IZUMO1-cfSGFP2 in cell-oocyte assay. *Izumo1-cfSGFP2* expressing COS-7 cells and oocytes were incubated for 2 h at 37°C with Mab18-Alexa546 at 0.5 μg/ml (red). Adherent COS-7 cells on the oocyte are indicated by asterisks. Nuclei were stained by Hoechst 33342. Inset shows middle DIC image among Z-stacks. Scale bar, 20 μm. The image of the oocyte is marked by dotted lines.

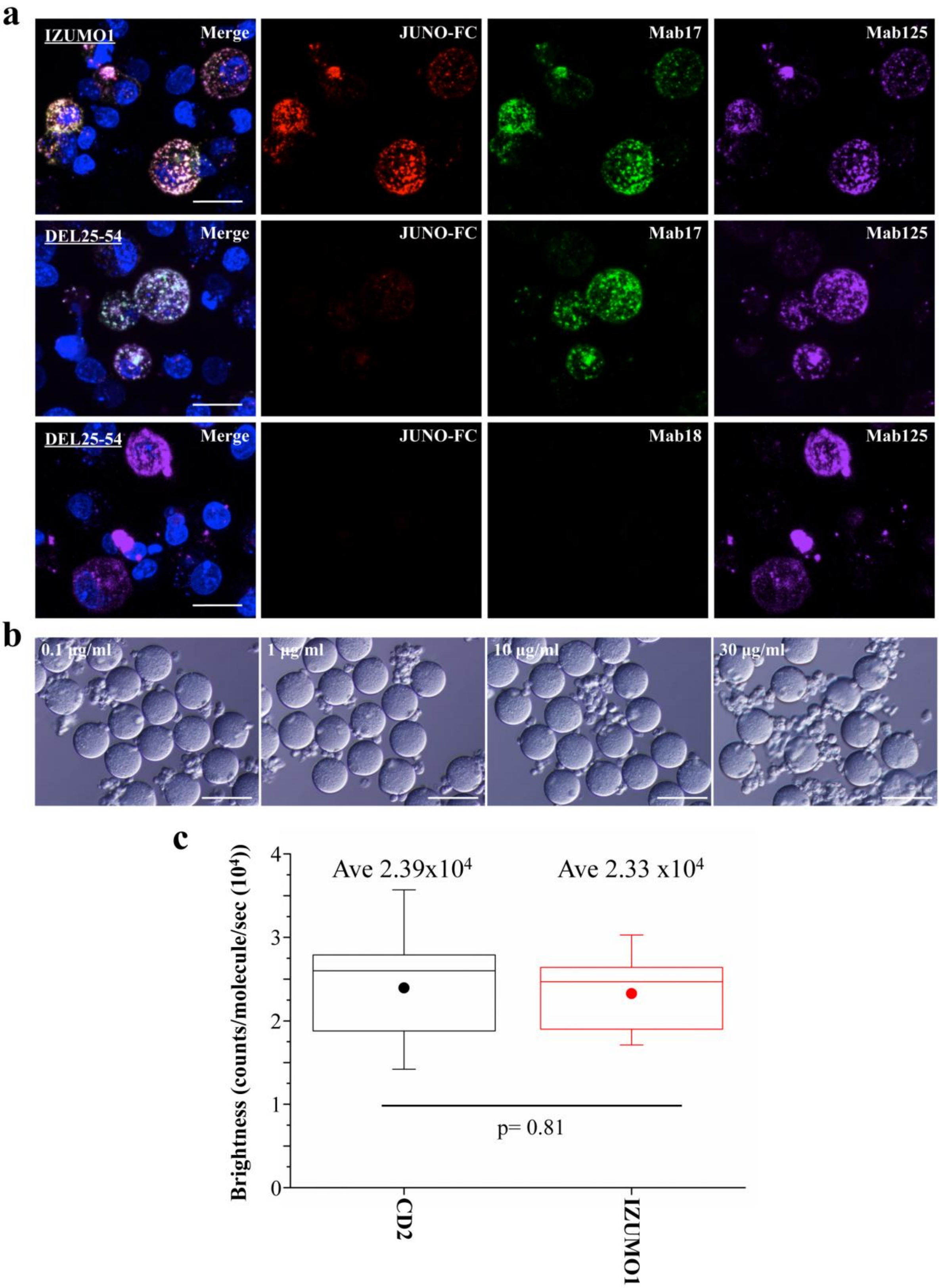

a
IZUMO1
Merge
JUNO-FC
Mab17
Mab125
DEL25-54
Merge
JUNO-FC
Mab17
Mab125
DEL25-54
Merge
JUNO-FC
Mab18
Mab125
b
0.1 µg/ml
1 µg/ml
10 µg/ml
30 µg/ml
c
Ave 2.39x10^4
Ave 2.33 x10^4
Brightness (counts/molecule/sec (10^4))
p= 0.81
CD2
IZUMO1
4
3
2
1
0

Supplementary Figure 3. Characterization of JUNO-FC binding and PCMH analysis of plasma membrane sheets.

(a) JUNO-FC lost binding ability to DEL25-54. COS-7 cells expressing *Izumo1* or DEL25-54 mutant were incubated with recombinant JUNO-FC labeled with anti–mouse IgG antibodies-Alexa546 at 1 μg/ml (red). Simultaneously, Mab17-Alexa488 (green; top and middle), Mab18-Alexa488 (green; bottom), and Mab125-Alexa647 (magenta) were co-incubated at 0.5 μg/ml for 1 h at 37°C. Binding of JUNO-FC was hardly detected in cells expressing DEL25-54. Nuclei were stained by Hoechst 33342. Scale bars, 20 μm.

(b) The effects of JUNO-FC on cell-oocyte inhibition. Recombinant JUNO-FC was incubated at various concentrations in *Izumo1* expressing cell-oocyte assays for 3 h at 37°C. Scale bars, 100 μm.

(c) PCMH of the plasma membrane sheets. Fluorescence intensity traces (total 1-2 × 10^5 counts) of the plasma membrane lysates prepared from CD2-mCherry expressing COS-7 cells, or IZUMO1-mCherry expressing COS-7 cells were recorded and the single molecule brightness was determined using PCMH. In this measurement, the average molecular brightness of CD2-mCherry was 23,900 ± 7,100 cpms (n=9) and that of IZUMO1-mCherry was 23,300 ± 4,700 cpms (n=9). A box chart is shown to indicate the ranges of 25-75% and 5-95% (bar) of each measurement. The average is marked by a dot. A t-test showed that there was no statistical difference in molecule brightness (p=0.81).

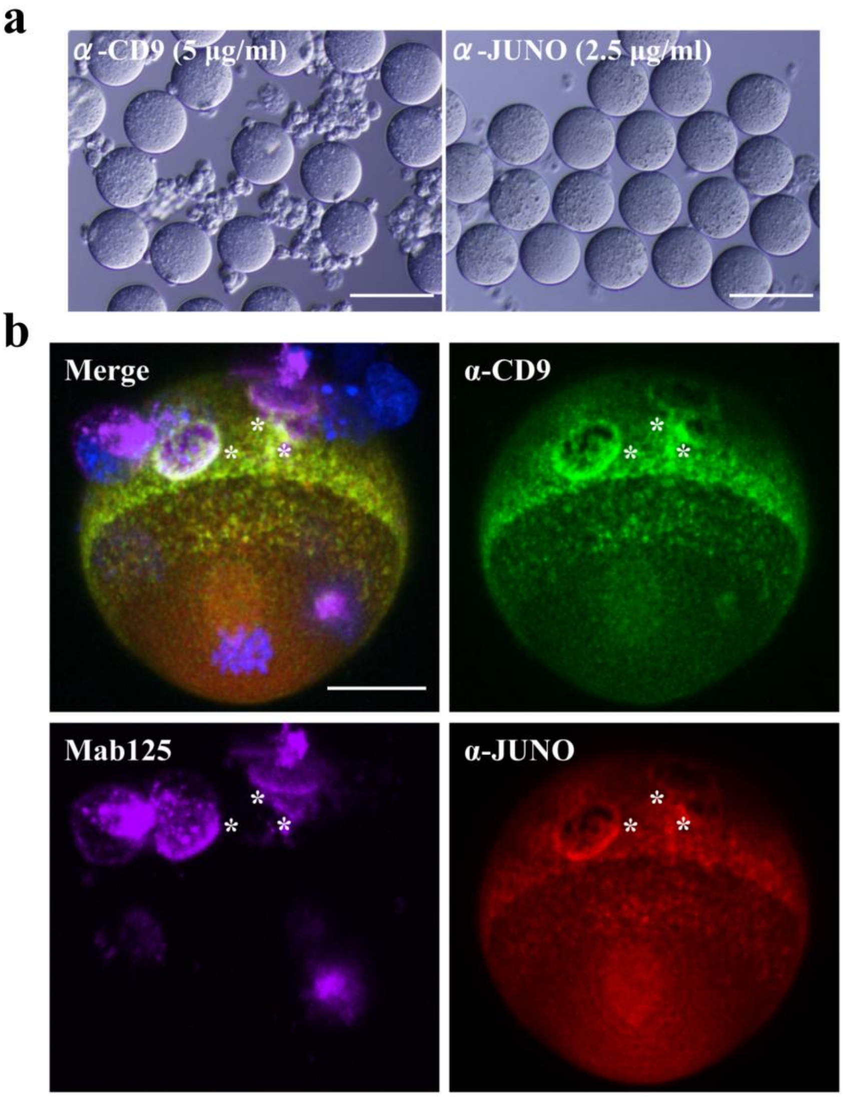

Supplementary Figure 4. The effects of JUNO antibody and JUNO distribution in cell-oocyte assay.

(a) The effects of specific antibodies for CD9 and JUNO. Antibodies against CD9 (MZ3) (5 μg/ml) or JUNO (TH6) (2.5 μg/ml) were incubated in a cell–oocyte assay for 3 h at 37°C. Anti-JUNO antibody completely inhibited cell-oocyte binding. Scale bars, 100 μm.

(b) Characteristic localization of JUNO and IZUMO1 in "touching" stage. Localization of JUNO (red), CD9 (green), and IZUMO1 (magenta) in a cell-oocyte assay was observed by each specific antibody, TH6, MZ3, and Mab125, respectively. The same methods as for Fig. 4a were applied. Asterisks indicate an adhered cell. Nuclei were stained by Hoechst 33342. Scale bar, 20 μm.

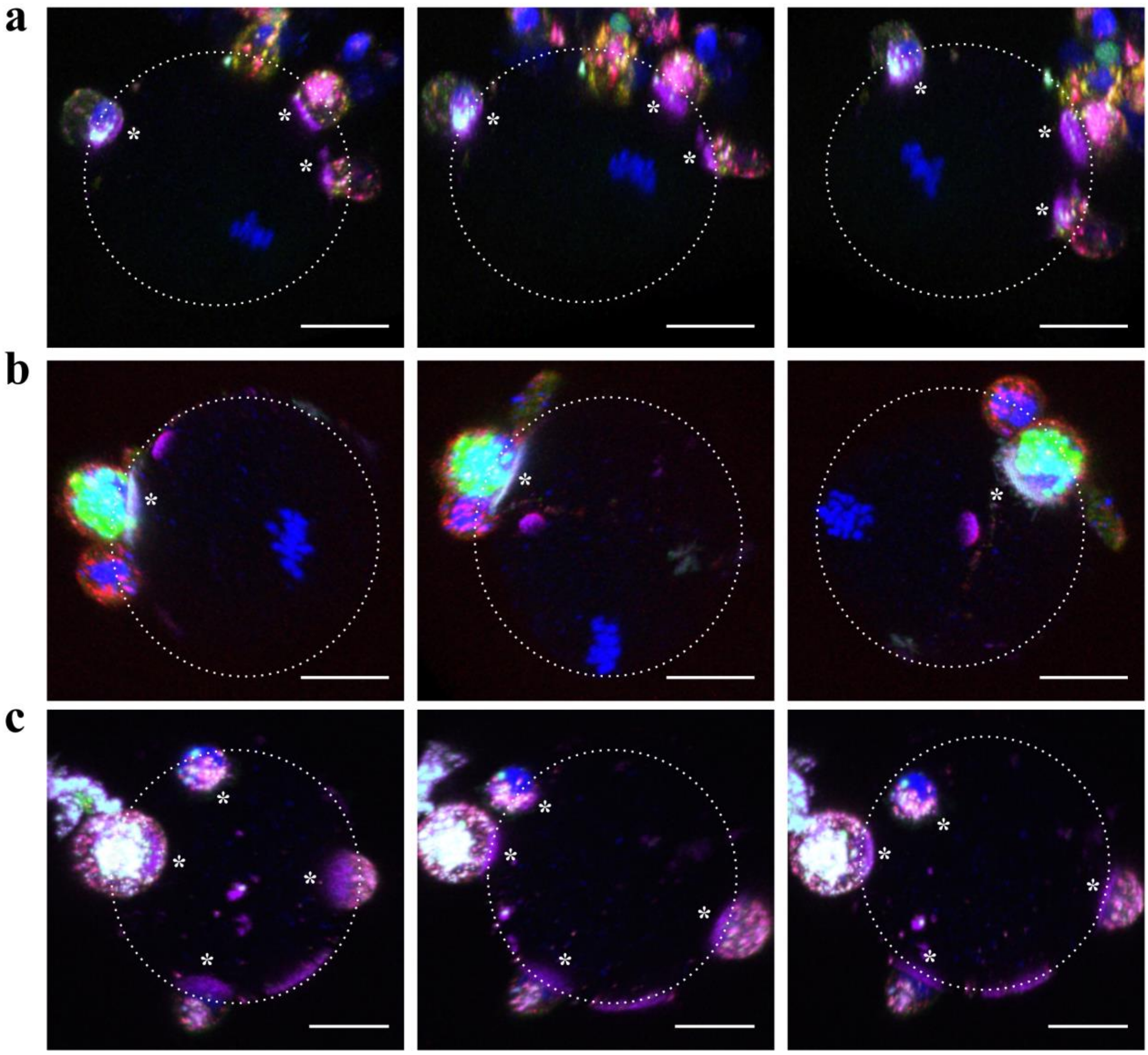

Supplementary Figure 5. Cell-oocyte assay photo from different angles.

(a) Corresponding to Fig. 1b

(b) Corresponding to Fig. 2c

(c) Corresponding to Fig. 3b

Asterisks indicate an adhered cell. Nuclei were stained by Hoechst 33342. Scale bar, 20 µm.
